# Clinical assessment of synbiotics for treating chronic kidney disease: A protocol for systematic review and meta-analysis

**DOI:** 10.1097/MD.0000000000022993

**Published:** 2020-12-04

**Authors:** Xiao-Jun Li, Yan-Yan Lu, Yuan Wang

**Affiliations:** Department of Nephrology, The Second Affiliated Hospital of Dalian Medical University, Dalian, Liaoning, P.R. China.

**Keywords:** chronic kidney disease, efficacy, safety, synbiotics

## Abstract

**Background:**

This research aims to evaluate the efficacy and safety of synbiotics for treating chronic kidney disease.

**Methods:**

Related articles written in English were sourced from EMBASE, PubMed, Cochrane Library, and China National Knowledge Infrastructure. These articles were used in the evaluation of the effect of synbiotics for treating chronic kidney disease. The extent of the relationship was assessed by calculating the pooled risk ratio, mean differences or standardized mean difference along with the equivalent 95% confidence intervals. The risk of bias introduced through each study was considered by adopting the Cochrane Risk of Bias Tool. Suitable statistical research methods were utilized for the synthesis of the data. The RevMan 5.3 software was used to conduct all statistical analysis.

**Results:**

The final results of the current study is due to be included in a peer-reviewed journal.

**Conclusion:**

The final remarks of the current study will be useful evidence for determining whether synbiotics is an effective and safe therapeutic method for treating chronic kidney disease.

**OSF registration number:**

DOI 10.17605/OSF.IO/UASF4 (https://osf.io/uasf4/).

## 1 Introduction

Globally, chronic kidney disease (CKD) remains a primary health concern.^[1]^ In 2019, the United States Renal Database System reported that, CKD is highly prevalent, which has been proved by the steady increase in the number of cases.^[2]^ CKD has been related with poor life standards and seriously adverse health outcomes, which includes infection, depression, failure of kidney which requires replacement therapy, and even fatal outcomes.^[3–5]^ Moreover, CKD is also closely related to the subsequent development and advancement of cardiovascular diseases, such conditions lead to high morbidity and fatalities.^[6]^

Recently, cumulative studies have demonstrated that gut dysbiosis and intestinal wall permeability can lead to kidney failure and cardiovascular risk through systemic inflammation and formation of uremic toxins, such as p-Cresyl sulfate, indoxyl sulfate, and trimethylamine N-oxide.^[7–9]^ The gut-kidney axis plays an important role in maintaining normal homeostasis, and during CKD progression, dysregulation of this axis occurs. Novel treatment methods targeted at restoring the symbiotic intestinal environment by utilizing prebiotics, probiotics, and synbiotics show potential as targeted therapeutic strategies to either delay or reverse the progression of the disease.

Presently, the outcomes of individual studies focused on studying the safety factor and effectiveness of synbiotics for the treatment of CKD have presented contradicting results. Moreover, there is no evidence of a systematic review that has assessed the safety factor and effectiveness of synbiotics for the treatment of CKD. Hence, this study aims to conduct a systematic review to evaluate the efficacy and safety factor of synbiotics for treating CKD.

## 2 Methods

This systematic review was listed under the Open Science Framework (OSF, https://osf.io/). The registration DOI number for which this study is listed is 10.17605/OSF.IO/UASF4. Moreover, the present review protocol aligns with PRISMA-P (Preferred Reporting Items for Systematic Review and Meta-Analyses Protocols) guidelines.

### 2.1 Eligibility criteria

#### 2.1.1 Types of studies

According to the plan, randomized controlled trials will be included in the present research. In accordance, other forms of studies, such as observational studies, non-randomized control studies, and case reports were excluded.

#### 2.1.2 Types of participants

People aged 18 years and above who have been diagnosed with CKD during any stage were included. Studies that involved participants that had contracted other forms of urinary system diseases, such as chronic pyelonephritis, ascending infection, and other chronic urinary diseases were excluded.

#### 2.1.3 Types of interventions and comparisons

The plan involved including randomized controlled trials of any synbiotics intervention, in comparison with placebo, no intervention, and other intervention method.

#### 2.1.4 Types of outcome measures

##### 2.1.4.1 Primary outcomes

Uraemic toxins; which includes phenols (p-Cresyl sulfate, p-cresol, and p-cresyl glucuronide) and indoles (indoxyl sulfate and indoleacetic acid).^[10]^

##### 2.1.4.2 Secondary outcomes

(1) Functions of the kidney; which includes blood urea nitrogen, glomerular filtration rate, creatinine clearance, and serum creatinine.
(2) Gastrointestinal functions; which includes improvement in gastrointestinal symptoms, transit time, and tolerance.
(3) Quality of life; can be assessed by adopting a validated scale, such as the Kidney Disease Quality of Life.
(4) Serious cardiovascular events; have been distinctly outlined by the investigator, includes heart failure, coronary artery disease, cerebrovascular disease, and myocardial infarction.
(5) All-cause mortality; including cardiovascular mortality and other infection-related mortality.

### 2.2 Search methods

#### 2.2.1 Electronic searches

The EMBASE, PubMed, Cochrane Library, and China National Knowledge Infrastructure were chosen as the databases to conduct a conclusive and thorough literature review to identify all studies that were potentially eligible. Regardless of the language and publication year of the articles, each database mentioned above was searched from inception to the present. Table 1 provides the search strategy for the Cochrane Library. In order to cater to additional electronic databases, the search terms are adapted appropriately.

**Table 1:** Search strategy for Cochrane Library (from inception to September 01, 2020).

Number	Search terms
#1	MeSH descriptor: [Kidney Diseases] explode all trees
#2	“kidney disease^∗^”:ti,ab,kw
#3	“renal disease^∗^”:ti,ab,kw
#4	“kidney failure”:ti,ab,kw
#5	“renal failure”:ti,ab,kw
#6	“kidney insufficiency^∗^”:ti,ab,kw
#7	“renal insufficiency^∗^”:ti,ab,kw
#8	(“CKD” or“CRD” or “CKF” or “CRF”):ti,ab,kw
#9	#1 or #2 or #3 or #4 or #5 or #6 or #7 or #8
#10	synbiotic^∗^
#11	MeSH descriptor: [randomized controlled trials] explode all trees
#12	“randomized controlled trial”:ti,ab,kw
#13	“controlled clinical trial”:ti,ab,kw
#14	(“randomised^∗^” or“randomized^∗^” or “placebo^∗^” or “randomly” or “trial^∗^”):ti,ab,kw
#15	#11 or #12 or #13 or #14
#16	#9 and #10 and #15

ab = abstract, CKD = chronic kidney disease, CKF = chronic kidney failure, CRD = chronic renal disease, CRF = chronic renal failure, kw = keywords, MeSH = medical subject headings, ti = title.

#### 2.2.2 Searching other sources

In order to ensure that related unpublished studies are not missed, the ClinicalTrials.gov (www.ClinicalTrials.gov) database is also included in the search for related studies that could be unpublished or not yet completed. Moreover, Google scholar, and the lists of references in the articles and all primary studies are also included in the search to identify grey literature.^[11]^

### 2.3 Data collection and analysis

#### 2.3.1 Selection of studies

**Figure 1:**
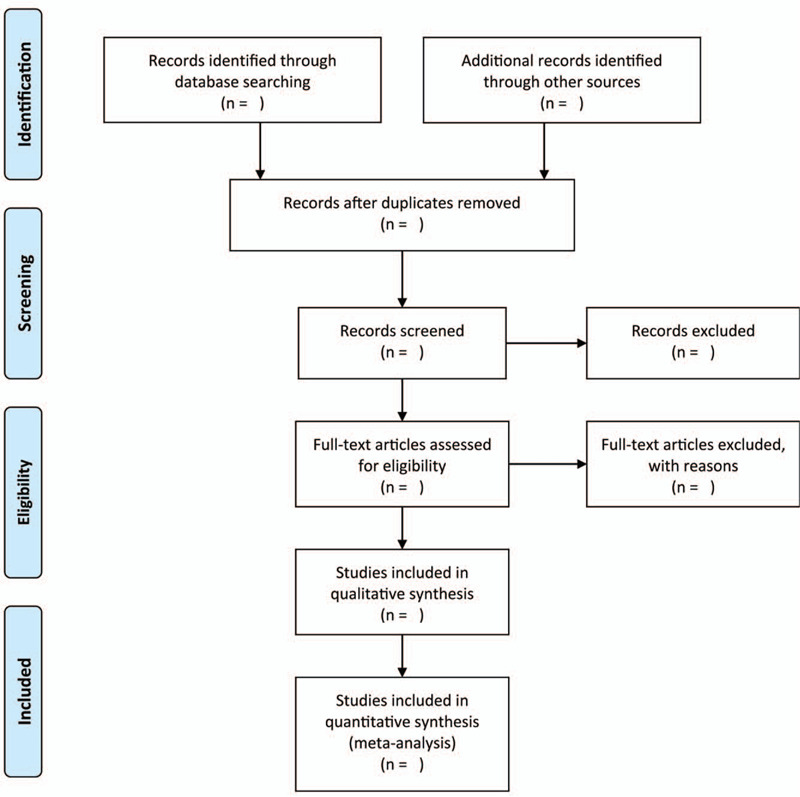
Flow diagram of the literature search.

The abstracts of related studies were independently checked by 2 reviewer authors. The relevance of the article was assessed, the reviewing authors acquired full trial reports of potential candidates for inclusion. Mutual consent was the primary method of resolving any form of disagreement between the authors, the alternative included recourse to a third review author. Studies that had full reports available were classified. However, studies that failed to satisfy the inclusion criteria were excluded. The PRISMA flow chart (Fig. 1) demonstrates the process of selecting studies.

#### 2.3.2 Data extraction

Two review authors independently extracted study data into a data extraction form to facilitate comparison of results. In the case of disagreements, a third review author resolved these disagreements through discussion. It was planned to record the tabulate the information into Excel spreadsheets under following classifications: author, publication year, design, sample size, the criteria of diagnostic, age, sex, eligibility criteria, details of treatment and control interventions, as well as outcome indicators.

#### 2.3.3 Assessment of risk of bias for included studies

According to plan, 2 review authors undertook the risk assessment of bias for each of the studies included by utilizing the criteria defined in the Cochrane Collaboration's tool, which includes 7 items, a rating of either high, low, or unclear risk of bias is given for each item. It was planned that any disagreement would be resolved through discussion.

#### 2.3.4 Measures of treatment effect

The aim was to present the treatment effect of dichotomous outcomes as a risk ratio with the corresponding 95% confidence intervals. For continuous outcomes, the data was planned to be presented as standardized mean difference or mean difference with the corresponding 95% confidence intervals.

#### 2.3.5 Assessment of heterogeneity

In accordance with the plan, standard Chi-squared statistic and *I*^*2*^ test were to be used for assessing the statistical heterogeneity amongst the studies that were included.^[12]^ The cases of *I*^*2*^ greater than 50%, or *P*-values less than .1 throughout the studies indicates a high heterogeneity, which was estimated with the aid of a random-effects model^[13]^; otherwise, the fixed-effects model was adopted.^[14]^

#### 2.3.6 Assessment of reporting biases

It was planned that the funnel plot and Egger test would be utilized for evaluating the potential publication bias if an excess of 10 trials are included in this systematic review.^[15,16]^

#### 2.3.7 Sensitivity analysis

The exclusion of studies containing high-risk or methodological data that lacks clarity, makes it possible to conduct sensitivity analysis to determine the stability and feasibility of the outcomes in this study.

#### 2.3.8 Subgroup analysis

Wherever applicable, it was aimed to perform the following subgroup analyses: various study characteristics, interventions, results, and demographic features (including age, gender, ethnicity, etc).

### 2.4 Ethics and dissemination

It is expected that the outcomes of the current study will be published in a peer-reviewed journal. Since private patient data is not obtained, it is not necessary to obtain ethical approval.

## 3 Discussion

Admittedly, studies that have been published previously have reported the use of synbiotics for treating CKD. However, contradicting results have led to controversial conclusions. Moreover, there has not been any prior instance of a systematic review been conducted to assess the effectiveness and the level of safety when using synbiotics to treat CKD. Hence, the present study will conduct a comprehensive assessment of the efficacy and safety of synbiotics in the treatment of CKD. The findings of this study could provide consistent outcomes that could be useful for clinically managing CKD.

## Author contributions

**Conceptualization:** Xiao-Jun Li.

**Funding acquisition:** Yan-Yan Lu.

**Investigation:** Xiao-Jun Li, Yan-Yan Lu.

**Methodology:** Xiao-Jun Li, Yan-Yan Lu, Yuan Wang.

**Software:** Xiao-Jun Li, Yan-Yan Lu.

**Supervision:** Yan-Yan Lu, Yuan Wang.

**Validation:** Yan-Yan Lu.

**Writing – original draft:** Yuan Wang.

**Writing – review & editing:** Yuan Wang.
